# Life course socioeconomic position and body composition in adulthood: a systematic review and narrative synthesis

**DOI:** 10.1038/s41366-021-00898-z

**Published:** 2021-07-27

**Authors:** Charis Bridger Staatz, Yvonne Kelly, Rebecca E. Lacey, Joanna M. Blodgett, Anitha George, Megan Arnot, Emma Walker, Rebecca Hardy

**Affiliations:** 1 Social Research Institute, Institute of Education, University College London, London, UK; 2 Department of Epidemiology and Public Health, University College London, London, UK; 3 Division of Surgery and Interventional Science, Faculty of Medical Sciences, Institute of Sport Exercise and Health, University College London, London, UK; 4 Department of Anthropology, University College London, London, UK

**Keywords:** Risk factors, Epidemiology

## Abstract

**Introduction:**

Multiple systematic reviews have investigated the relation between socioeconomic position (SEP) and body mass index (BMI) throughout the life course. However, BMI does not capture quantity and distribution of fat and muscle, which are better indicators of obesity than BMI, and have been independently linked to adverse health outcomes. Less is known about the relation between SEP and body composition, and the literature has not been reviewed. We therefore systematically reviewed the literature on the association between life course SEP and body composition in adulthood.

**Methods:**

A protocol was registered on PROSPERO (CRD42019119937), and the review followed PRISMA guidelines. An electronic search of three databases (MEDLINE, Embase Classic + Embase and SPORTDiscus) was conducted. Original studies in the English language were included that examine the association between any recognised measure of SEP at any age and body composition (fat mass, fat-free mass, ratio and distribution) in adulthood, measured using a direct technique, i.e., not an anthropometric measure. A narrative synthesis was conducted.

**Results:**

A total of 47 papers were included in the final review, none were from low-income countries (LICs). Greater advantage in childhood and adulthood was associated with lower fat levels in high-income countries (HICs). Associations in the opposite direction were found exclusively in middle-income countries (MICs). No studies in MICs reported associations for childhood SEP. For measures of lean mass, the majority of papers reported no association, or greater advantage in adulthood associated with higher lean mass, with little variation between HICs and MICs. Associations in HICs are more often observed in women than men.

**Conclusion:**

The results indicate that fat measures follow similar patterns to those seen for BMI, and that women in HICs are more likely to experience inequalities in both fat and lean measures. Further research in LICs and MICs is needed.

## Introduction

Socioeconomic position (SEP) across the life course has been repeatedly linked with obesity in high-income countries (HICs) and has been the subject of multiple systematic reviews [1–6]. These reviews demonstrate predominantly that, among adults in HICs, advantaged SEP in both childhood and adulthood is associated with lower levels of obesity defined by anthropometric measures such as body mass index (BMI), with stronger associations in women [1–3, 5, 6]. In studies from middle-income countries (MICs), systematic reviews have generally found associations in the same direction among women, but less consistent associations among men. In those from low-income countries (LICs) associations have been found in the opposite direction such that more advantaged SEP is associated with higher rates of obesity [7].

SEP is an umbrella term for multiple measures of social and economic circumstances that influence an individual’s position in society [8]. Different measures of SEP capture different aspects of these circumstances and may relate to health outcomes in different ways, thus providing information about the underlying pathways. Education is a proxy for health literacy; occupational social class represents working conditions and social standing; income is a direct measure of material resources; and area-level SEP captures elements of the broader environment. However, in LICs and MICs a composite measure of material living standards may better capture circumstances than education, occupation or income.

Although the literature linking SEP and BMI has been extensively reviewed, the evidence linking SEP and body composition has not. BMI is a measure of weight for height that does not distinguish fat mass (FM) from fat-free mass (FFM). Measures of body composition provide estimates of the proportion of FM to FFM, including in some cases lean mass (LM)—a measure of FFM most often captured through dual x-ray absorptiometry (DXA), which excludes bone mass—and can inform about the location of FM [9].

A lower proportion of FM and higher proportion of FFM has been shown to be associated with a reduced risk of cardiovascular disease and total mortality [10, 11]. Distribution of FM is also associated with cardiovascular and metabolic disease, with higher central adiposity and higher android-to-gynoid FM ratio shown to increase risk [12–15]. In addition, FFM plays a role in development of insulin sensitivity, with skeletal muscle being a key sight of glucose uptake [16]. Having higher levels of FFM therefore has the potential to reduce and delay the onset of metabolic disorders [17, 18]. LM is also important for maintaining musculoskeletal health in older age, with sarcopenia characterised by loss of skeletal muscle and muscle strength. Lower levels of LM also increases risk of osteoporosis as LM is positively associated with bone mineral density [19]. If inequalities in FM are similar or stronger than the inequalities in BMI, while greater advantage is associated with higher LM, then the impact of inequalities in adiposity on health may be underestimated if based on BMI alone.

We aimed to perform a systematic review of the literature to assess the association between SEP and measures of body composition in the general population. In addition, we aimed to assess differences in socioeconomic inequalities in body composition by birth cohort, sex and by SEP measure. In this paper we focus on associations between (a) SEP measured in childhood and adult body composition and (b) SEP in adulthood and adult body composition.

## Methods

The protocol for this review was registered with the PROSPERO database (CRD42019119937) and has been carried out using the Preferred Reporting Items for Systematic Reviews and Meta-Analyses (PRISMA) checklist (Supplementary File 1). Full details of the methods can be found in the published protocol [20].

### Eligibility

Peer-reviewed papers in the English language reporting an association between any recognised indicator of SEP (e.g., income, education, overcrowding, area-level deprivation) and a direct measure of body composition (i.e., measured using bioelectrical impedance analysis (BIA) or DXA) were included. Observational studies including samples from the general population were included where body composition was measured at the same, or later, time point to SEP. Body composition was defined as any measurement related to total FM and FFM, location of FM and FFM or any proportion or ratio of measures of FM and FFM. Studies based on anthropometric measurements such as BMI or abdominal circumference were excluded.

### Search strategy

An electronic search of three databases (MEDLINE and Embase Classic + Embase using OvidSP as the interface, SPORTDiscus using EBSCO as the interface) was conducted by CBS to identify appropriate studies published from the earliest entry available for each database (MEDLINE: 1879; Embase Classic + Embase: 1947; SPORTDiscus: 1892) up until the 30th January 2019. The search terms used are shown in Table 1. The search was broad and included body composition in childhood as well as in adulthood. The results of the search were de-duplicated and stored in the reference manager, Endnote. This database was exported to Rayyan QCRI [21] to conduct screening. Titles and abstracts were screened for eligibility and full texts of the eligible papers were subsequently screened by CBS, AG, and JMB. Reasons for exclusion of studies were recorded at the full-text screening stage. The reference list of eligible full texts was screened to identify additional papers.

**Table 1:** Search terms.

Search terms	
Database	Medical Subject Headings (MeSH) terms
Medline	Body composition: exp Body Composition/; Adipose Tissue/; exp Body Fat Distribution/; Obesity/or obesity; abdominal/
	Body composition measures: Electric Impedance/; Magnetic Resonance Imaging/; Tomography, X-Ray Computed/; Densitometry/; Whole-Body Counting/; Plethysmography/
	Socioeconomic position: socioeconomic factors/ or poverty/ or poverty areas/ or social class/; Educational status/ or income/ or occupations/ or social conditions/
Embase + Embase Classic	Body composition: Body composition/ or body distribution/ or body fat/ or body fat distribution/; Obesity/; lean body weight/; Fat mass/
	Body composition measures: Impedance/; nuclear magnetic resonance imaging/; computer assisted tomography/; densitometry/; whole body counting/; Total body water/; plethysmography/
	Socioeconomic position: socioeconomics/ or educational status/ or income group/ or poverty/; income/ or occupation/ or household income/; social status/ or social background/ or social class/; education/;
SPORTDiscuss	Body composition: ((DE “BODY composition” OR DE “HUMAN body composition”) OR (DE “OBESITY”)) OR (DE “ADIPOSE tissues”)
	Body composition measures: ((((DE “BIOELECTRIC impedance”) OR (DE “COMPUTED tomography”)) OR (DE “MAGNETIC resonance imaging”)) OR (DE “BONE densitometry”)) OR (DE “PLETHYSMOGRAPHY”)
	Socioeconomic position: ((DE “EDUCATION”) OR (DE “EDUCATIONAL attainment”)) OR (DE “HEALTH & income”)
*Free text search terms*	
Body composition	1. Body Composition MeSH Terms
	2. (Body adj3 (composition or distribution))
	3. ((fat or adipos*) adj3 (composition or distribution or mass or index or kg or total))
	4. ((muscl* or lean) adj3 (composition or distribution or mass or index or kg or total))
	5. ((fat-free) adj3 (mass or kg or total))
	6. ((android or gynoid or visceral or appendicular or abdominal or intra-abdominal) adj3 (fat or lean or muscle or mass or adipos*))
	7. 1 OR 2 OR 3 OR 4 OR 5 OR 6 Body composition Measures
Body composition measures	8. Body Composition Measures MeSH Terms
	9. ((impedance) adj3 (bioelectrical or foot-to-foot or hand-to-foot or analy?is))
	10. (bioimpedance or body fat analy?er or body composition analy?er or tanita)
	11. (dual x-ray absorptiometry or DEXA or DXA or dual-energy X-ray absorptiometry)
	12. (magnetic resonance imaging or MRI)
	13. (Computed tomography or CT or CAT scan)
	14. (densitometry)
	15. ((neuron activation or total body counting or whole body counting))
	16. (total body water)
	17. (air-displacement plethysmography)
	18. 8 OR 9 OR 10 OR 11 OR 12 OR 13 OR 14 OR 15 OR 16 OR 17
	19. 7 AND 18
Socioeconomic position	20. Socioeconomic Position MeSH terms
	21. (social class or social status or social position or socio-economic or socioeconomic or social circumstance*)
	22. (sociodemo*)
	24. Educat*
	25. (income* or manual or class)
	26. (depriv* or poverty or overcrowding)
	27. 20 OR 21 Or 22 OR 23 OR 24 OR 25 OR 26
	28. 19 AND 27, 29. Limit to English Language (and Human in OvidSP)

MeSH terms are main heading descriptor terms available in each database and are determined by the indexing method adopted by each database. Free text search terms were entered into all databases, along with the results of the database-specific MeSH terms.

*P < 0.05.

**P < 0.01.

***P < 0.001.

### Extraction and quality assessment

Relevant data that examined the association between at least one measure of SEP and a measure of body composition were double extracted by CBS, AG, JMB, MA and EW using a data extraction form. Data extracted included citation details (author, title, paper, publication year, publication type), study details (cohort or sample description, study design, country, participant numbers), participant details (birth year or age or participants, sex of participants), exposure and outcome details (type of SEP and body composition variables presented, age variables recorded, how the variables were ascertained and measured) and statistical methods and information on adjustment for potential confounders and mediators. All available statistics relating to the association under study were extracted, along with statements of direction in text where statistics were not presented.

Assessment of study quality was carried out, using an amended version of the Newcastle-Ottawa Quality Assessment scale [22]. Quality assessment was used to inform on the variability of quality across the papers and potential bias arising, and not to guide the inclusion of results into the review. The original quality assessment form was amended during the review to account for the large number of cross-sectional studies and the variability in statistical reporting (questions 3bi, 3bii and 4—Supplementary File 2). Google Form was used to aid extraction and WebPlotDigitizer (https://automeris.io/WebPlotDigitizer/) was used to extract data that were only presented in graphs. Two reviewers (CBS and either AG, JMB, MA or EW) worked independently to complete every stage of the screening, extraction and quality assessment process. Disagreements were resolved through discussion.

### Synthesis

Due to the considerable variability in analytic methods and presentation of results, a meta-analysis was not possible, and nor was it possible to use a funnel plot to assess publication bias. A narrative synthesis was conducted, guided by the Economic and Social Research Council Methods Programme guidelines [23]. Synthesis has been conducted according to the groupings of: (a) childhood SEP and adult body composition and (b) adult SEP and adult body composition.

The majority of papers presented multiple associations. Therefore, similar to methods adopted by McLaren [3] and Ball and Crawford [24], the individual associations between each different SEP and body composition variable, rather than individual papers, were considered as the units of analysis.

Associations were classified into positive associations (those reporting greater socioeconomic advantage associated with higher body composition measure), inverse association (those reporting greater socioeconomic advantages associated with lower body composition measures), non-linear (e.g curvilinear or heterogenous) association and no association. Associations were assigned to groups according to the effect estimates and 95% confidence intervals. Where this information was missing, trends identified in descriptive data, *P* values or statements of direction were used.

Where associations were provided for multiple subgroups, primary results selected for summary were those in men and women included together. As analysis by sex was pre-specified in the protocol, where papers only reported in males and females separately, both associations were included in the primary summary of results.

Heterogeneity was explored by body composition measure (FM, FFM, ratio and distribution), birth cohort, sex and SEP measure. On extraction it became clear that country income level should also be considered as sources of heterogeneity. Studies were categorised into HICs, upper middle- and lower middle-income countries, according to the World Bank classification in 2019 [25]. Those papers from ‘upper middle’ and ‘lower middle’ income countries will all be referred to as ‘middle-income countries’ (MIC).

## Results

**Fig. 1:**
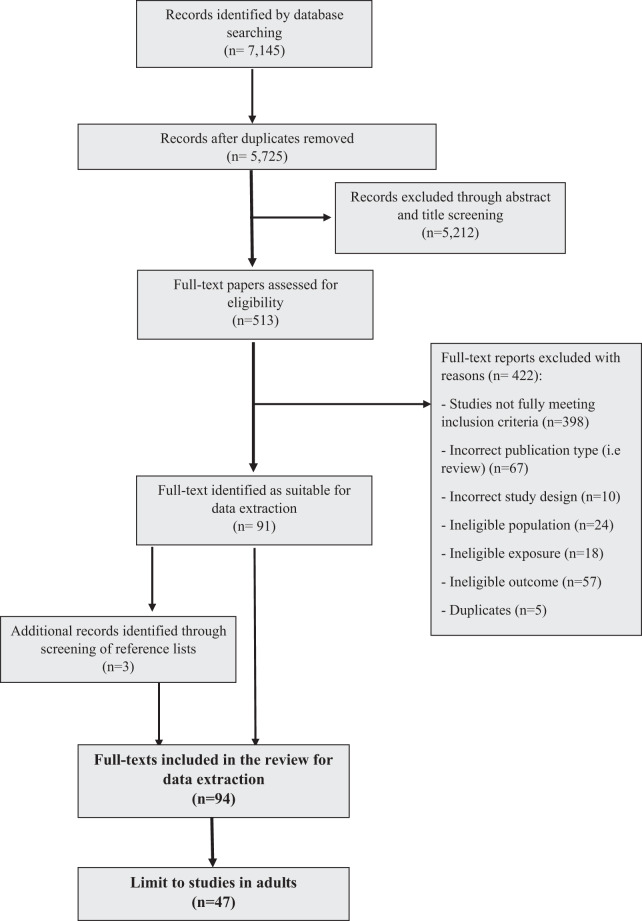
Study selection process outlined with PRISMA flowchart. Numbers given for reasons for exclusion during full-text screening stage equal more than the total excluded at this stage (*n* = 422), because reasons for exclusion are not mutually exclusive.

Figure 1 shows the study selection process, as outlined in the PRISMA flowchart. Searching the databases for potential papers returned 7145 papers, with 5725 once duplicates were removed. Title and abstract screening resulted in 513 papers, with 91 papers remaining following full-text screening. Searching the reference lists for additional papers returned 3, bring the total included papers to 94. Of those, 47 investigated either adult or childhood SEP and adult body composition and are reported on here [26–72]. Descriptive results and quality assessment for the included papers are shown in Table 2. The majority (*n* = 27) of papers were rated as medium quality, 14 were rated as low quality, and 6 papers were rated as high quality.

**Table 2:** Descriptive characteristics of included studies.

First author	Year published	Country	Data set used (if named)/description of sample	*N*	SEP measures	Body composition measures	Technique	Age range (mean/median)	Quality assessment
Agha^a^ [26]	2013	USA	LEAP	400	Prenatal SEI (a composite score using a weighted percentile of both parents educational attainment, occupation and income relative to the US population)	Android Fat Mass; Android-to-Gynoid Ratio; Trunk-limb Ratio	DXA	Mean: 48 SEP measured prenatally	6*
Al-Qaoud [27]	2011	UK	Whitehall II	5533	Occupation	FFM; LMI	BIA	55–79 (mean: 66)^c^	5^*^
Amador [28]	2017	Scotland	Scottish Family Health Study	11,118	Socioeconomic Covariates (SIMD, years of education, household size, vehicle ratio and job status); SIMD; Education	FM	BIA	18–98	2^*^
Amani [29]	2007	Iran	Healthy married women who had been to one of the 14 main city health centres for a periodic child check-up	637	Education	%FM	BIA	18–40 (mean: 27)	2^*^
Azarbal [30]	2016	USA	Women’s Health Initiative Clinical Trial and Observational Study	8832	Income; Education	LM	DXA	50–79 (mean: 80)	4^*^
Bae^a^ [31]	2018	Korea	KHANES	3837	Education	LM, FBF	DXA	50+	2^*^
Bai [32]	2016	China	Men and women recruited through printed advertisement from the health survey centre of Shanghai Huadong Hospital	415	Education	ASM; FFM	BIA	60–100 (mean: 72)	3^*^
Bann [33]	2014	UK	NSHD	1558	Paternal Occupational Class; Maternal Education; Paternal Education; Own Education; Own Occupational class; Household Income	FMI; Android-to-Gynoid Ratio; ASMI	DXA	60–64 SEP collected multiple times from age 4	8^*^
Barrera [34]	2017	Chile	Independently living older women in metropolitan Santiago belonging to community centres for older people	86	Education	FM; FFM	DXA	Mean: 73	1^*^
Beydoun [35]	2009	USA	HANDLS	1227	SES (a single measures on a standardised z score scale obtained through PCA of education and PIR)	Trunk FM; Trunk FM as % of Body Fat; Total body FM	DXA	30–64	7^*^
Bhupathiraju [36]	2011	USA	Boston Puerto Rican Osteoporosis Study	629	Education; Income	Abdominal Fat	DXA	Mean: ~60	7*
Brennan [37]	2009	Australia	Geelong Osteoporosis Study (GOS)	1043	Area based SES (Socio-Economic Indexes For Areas (SEIFA) value generated based on the 2006 Census for each subject)	FM; LM	DXA	20–92 (mean: 49)	6*
Buemmann [38]	1995	Canada	Quebec Family Study	726	Education	%FM	Underwater Densitometry	Means: 42–46	4*
De Marchi [39]	2012	Brazil	Random sample of South Brazilians	471	Income; Education	%FM^b^	BIA	60–80+	7*
Dugan [40]	2010	USA	SWAN	369	Education	Intra-Abdominal Fat	CT	Mean: 51	5*
Dupuy [41]	2013	France	EPIDOS	1989	Education	ASM^b^	DXA	Mean: 80	6*
Fedewa [42]	2014	USA	First year college students recruited through email and print advertising	177	Area-level SES (Area-level deprivation index)	%FM	DXA	18–20 (mean 18)	3*
Guo [43]	2018	UK	Biobank	162,691	Area-level SES (Townsend deprivation index)	FM	DXA	40–70 (mean 59)	3*
Kazlauskaite [44]	2012	USA	SWAN	257	Income	IAT; IAT-SAT ratio	CT and DXA	Mean: 52	6*
Keighley [45]	2006	Samoa and American Samoa (USA)	Samoan Family Study of Overweight and Diabetes	1711	Education; Material Lifestyle; Occupation	%FM	BIA	2 age groups: 18–44, >45 (max age 90)	4*
Keino^a^ [46]	2017	Kenya	Random selection of women age 15–45 in Kenya	Not reported	Education	%FM; FMI	Deuterium oxide dilution solution (total body water)	15–45	2*
Kim [47]	2015	Republic of Korea	KHANES	3285	Education; Income	ASM^b^	DXA	65+	4*
Krueger^a^ [48]	2010	USA	MIDUS	211	Education	LM; FM	DXA	38–86 (mean 54)	1*
Kruger [49]	2016	South Africa	PURE	247	Education	ASMI^b^	DXA	45+ (mean 57)	4*
Kulkarni [50]	2010	India	Adult women who were not pregnant or lactating, residing in a large urban slum (Addagutta) in Hyderabad	278	Type of Employment	LM; FM; Leg FM; Trunk FM	DXA	Mean: 41	5*
Lahmann [51]	2000b	Sweden	Malmo ¨ Diet and Cancer study	27,808	Occupation	%FM	BIA	45–73 (mean: 57–59)	7*
Lahmann [52]	2000a	Sweden	Malmo ¨ Diet and Cancer study	5464	Education; Employment; Occupation; Parental Occupation	%FM	BIA	45–73 Parental occupation recalled	6*
Lantz [53]	2008	Sweden	Random selection of adolescents from population register from industrial town Trollhättan, Sweden	106	Fathers Education	FM; LM; %FM; %LM	DXA	20.5	6*
Lewin [54]	2014	France	RECORD	4078	Own Education, Own Employment, Parental Education; Financial Strain; Neighbourhood Education Level	FMI; %FM	BIA	30–79 Childhood SEP variables recalled	7*
Lewis [55]	2009	USA	SWAN	418	Education	Visceral Fat	DXA	42–62 Those in original SWAN cohort has SEP measured at baseline (age 31–56)	6*
Loucks [56]	2015	USA	New England Family Study	394	Childhood SEI (weighted percentile of both parents’ educational attainment, occupation, and income relative to the US population); Education	Android Fat	DXA	46–48 (median: 47) Childhood SEP from age 7	4*
Lourenco [57]	2008	Brazil (Amazonia)	Adults from Suruı´ population, an indigenous society from the Brazilian Amazon	188	SES (based on: materials used in house building; number of sleeping rooms; presence of modern household appliances; and, presence of western style furniture)	%FM	BIA	20–85 Grouped into those 20–49.9 and 50+	4*
McClure [58]	2011	USA	SWAN	301	Financial Strain; Education	Visceral Fat	CT	46–58 (means: 50–51), SEP measured at baseline interview (age 42–52)	6*
Mongraw-Chaffin [59]	2017	USA	MESA	1910	Education; Income	Visceral Fat	CT	45–84 (mean 65)	4*
Özener [60]	2007	Turkey	Sample of males made up of labourers from low SEP, non-labourers of low SEP and non-labourers of high SEP living in Ankara, Turkey	309	SEP (determined by occupation (labourer or student), type of schooling (i.e., private schooling or vocational training) and area of city abide in for school and work)	FM; %FM; FFM; FMI; FFMI	BIA	17–20 (mean 18)	1*
Pirila [61]	2012	Finland	Sample taken from birth cohort of full-term infants with a birth weight over 3000 g, born at the Helsinki University Central Hospital between January and March 1975	158	Education; Fathers Education	z-%FM^b^; LM; %Trunk Fat	DXA	32, childhood SEP variables recalled	6*
Powell [62]	2016	Italy	Participants selected from ongoing cohort (Milan), followed at the International Centre for the Assessment of Nutritional Status (ICANS, University of Milan)	3341	Education; Occupation	VAT:FFMI^b^; FM:FFM^b^	Abdominal ultrasonography and BIA	18–81 (mean 46)	3*
Rangel Peniche [63]	2018	Mexico	Non-random sample of health adults from two regions of Mexico	430	Income; Employment Status; Education	ASM; ASMI; FM; FMI; %FM	DXA	60–83 (means: 69–72)	2*
Rebato [64]	2001	Spain	Caucasian adults living in marginal districts of Bilbao, Spain	446	SES (low SES determined from degree of poverty and marginality (i.e., homelessness, receiving state assistance))	%FM	BIA	18–65	3*
Sallinen [65]	2011	Finland	Health 2000 Survey	2139	Education; Income	%FM^b^	BIA	55+ (mean 67)	5*
Seppanen-Nuijten [66]	2009	Finland	Health 2000 Survey	5789	Education	FFM^b^	BIA	30+ Split into those aged 30–64 and 65+	6*
Sotillo [67]	2007	Spain	Probabilistic and stratified sample of adults in the region of Andalusia	394	Education	FFM; %FM; FM	BIA	20–60 (means: 42–44)	6*
Suder [68]	2009	Poland	Young working males employed by the Sendzimir Metallurgical Plant in Cracow, and other companies on its premises	259	SES (based on birthplace, place of residence in childhood, social class, education level, type of work)	%FM	BIA	20–30 (mean 27)	3*
Velasquez-Melen [69]	2005	Brazil	Women of good health with no chronic or acute metabolic or infectious complaints, recruited from municipal health centre in the city of Belo Horizonte	410	Education; Income	%FM	BIA	20–55 (mean 33)	4*
Visser [70]	1998	USA	Framingham Heart Study	753	Education	LM^b^; %FM^b^	DXA	72–95 (mean 76–79), Baseline measures taken 30–62	6*
Wu [71]	2003	Taiwan	Tainan Diabetes and Related Chronic Disease Survey	1103	SES (Hollingshead index)	%FM	BIA	20+ (mean 48)	5*
Yliharsila [72]	2007	Finland	Helsinki Birth Cohort	2003	Childhood Social Class (based on fathers occupation); Social Class (based on own occupation)	%FM	BIA	Mean: 62, SEP derived from census data from 23 years earlier and multiple points in childhood	6*

Where papers have reported either body fat or fat mass, the variable is listed as just fat mass.

*SEI* Socioeconomic Index, *SIMD* Scottish Index of Multiple Deprivation, *SEP* socioeconomic position, *SES* socioeconomic status, *PIR* Poverty Income Ratio, *FM* fat mass, *FFM* fat-free mass, *FMI* Fat Mass Index, *FFMI* Fat Free Mass Index, *ASM* Appendicular Skeletal Muscle, *ASMI* Appendicular Skeletal Muscle Index, *LM* lean mass, *LMI* Lean Mass Index, *IAT* intra-abdominal adipose tissue, *SAT* subcutaneous abdominal adipose tissue, *VAT* visceral adipose tissue, *BIA* Bioelectrical Impedance Analysis, *DXA* dual x-ray absorptiometry, *CT* computed tomography, *PCA* principle component analysis, *TAPS* Tsimane’ Amazonian Panel Study, *MESA* The Multi-Ethnic Study of Atherosclerosis, *SWAN* The Study of Women’s Health Across the Nation, *RECORD* Residential Environment and Coronary Heart Disease Cohort Study, *PURE* Prospective Urban and Rural Epidemiology, *MIDUS* The Midlife in the United States, *KNHANES* The Korea National Health and Nutrition Examination Survey, *EPIDOS* EPIDémiologie de l’OStéoporose, *HANDLS* Healthy Aging in Neighbourhoods of Diversity across the Life Span, *NSHD* National Survey of Health and Development, *LEAP* Longitudinal Effects on Aging Perinatal Project.

* indicates the star rating on the quality assesment.

^a^Indicates abstract only. Mean ages are rounded to one full year. Range of mean age is given when only the means of subgroups, and not the full samples, are presented (i.e., mean ages presented separately for males/females).

^b^Papers have created a categorical or dichotomous variable (i.e., underfat/normal fat/excess fat) based on the indicated continuous measure.

^c^For those individuals who had retired at phase 9, an earlier measure of SEP was taken.

### Characteristics of included studies

There were 40 distinct study samples across the 47 papers, published between 1995 and 2018. The Study of Women’s Health Across the Nation (SWAN) was used in four papers. There were four other samples used in two papers each: Health 2000 Survey; Malmo ¨ Diet and Cancer study; The Korea National Health and Nutrition Examination Survey (KHANES); and the New England Family Study was used in addition to the Longitudinal Effects of Ageing on Perinatal (LEAP) project, which is a sub-set of the New England Family Study. The majority of papers were from Europe (*n* = 18, 38%) or North America (*n* = 14, 30%). There were 13 studies conducted in samples from the US and four each in Finland and the UK. Eleven papers were in MICs. No studies from LICs were identified. Sample sizes ranged from 86 to 162,691, with the interquartile ranges between 309 and 3285. One paper did not report sample size.

There was substantial variability in body composition measures used, especially for fat-free measures, but also terminology used to describe them. The different measures identified, and the definitions adopted in this review are shown in Supplementary Table 1. For the purposes of the review, fat-free measure is a general term that will refer to all measures of body composition that are not FM measures. Lean body mass (LBM) is a fat-free measure equivalent to FFM plus essential fats found in the nervous system, cell membranes and bone marrow [73]. This is different to the measure of LM, which is equivalent to FFM minus bone mass, and more closely captures what is colloquially thought of as ‘muscle’ [74]. LBM and FFM are most often measured through BIA, as it is not able to separate bone mass and lean tissue, whilst LM is most often measured through DXA as it uses a three-compartment model that splits the body into fat, lean and bone mass [74]. Appendicular skeletal muscle (ASM) is included as a total body fat-free measure, as muscle mass in the limbs represents 75% of total skeletal muscle mass (SMM) [74, 75]. The majority of papers used DXA to measure body composition (*n* = 22), with BIA the next most common method (*n* = 20). The remaining studies used underwater densitometry (*n* = 1), CT scans (*n* = 4), deuterium oxide dilution solution (total body water) (*n* = 1) and abdominal ultrasonography (*n* = 1). Two papers used more than one method.

Fat measures were considerably more frequently reported (in 30 papers) than fat-free measures (in 20 papers), with percentage fat mass (FM%) the most frequently analysed (reported in 21 papers). LM was the most frequently used fat-free measure (in 8 papers).

The most frequently reported SEP variable was education (in 32 papers). ‘Composite SEP’, which we define as any index combining two or more individual-level SEP indicators, was used the same number of times as income (10 papers). Occupational social class was used in 7 papers, and area-level SEP, which we define as any measure that captures the deprivation of an area, in 5 papers.

The majority of papers were conducted in adults over 45 or in samples with a mean age over 45 (*n* = 29). There were 13 studies where participants were either aged over 60 or had a mean age of 60+. Few cohort studies provided birth year (*n* = 5), or information from which this could be calculated, preventing assessment of secular differences in body composition by birth cohort as specified in the protocol.

### Childhood socioeconomic position and adult body composition

There were seven papers investigating the associations between childhood SEP and adult body composition. We were unable to assess heterogeneity due to the small number of papers, all of which were conducted in HICs. Results are summarised in Table 3.

**Table 3:** Direction of association reported between socioeconomic position in childhood and body composition in adulthood, presented in narrative form.

Paper	Country	*N*	SEP measure and time of measurement	Body composition measure and age	Findings
Agha (2013) [26]	USA	F: 228 M: 172	Prenatal SEI	Android FM, Android-to-Gynoid Ratio, Trunk to Limb FM Ratio Age: 48	Inverse associations between prenatal SEI and android FM, android-to-gynoid ratio and trunk to limb FM ratio in females with adjustment for age, race and maternal variables. No association in males
Bann (2014) [33]	UK	M: 746 F: 812	Occupational Social Class (age 4) Mothers and Fathers Education (age 6)	FMI, ASMI, Android-to-Gynoid Ratio Age: 60-64	All three childhood SEP measures were significantly (*P* < 0.05) related to FMI and android-to-gynoid ratio in men and women, and significantly associated with appendicular lean mass index in men before adjustment for fat mass, but not in women. Following adjustment for fat mass, the association with appendicular lean mass index was significant in women but not men. Own education and adult SEP may explain part of the association between paternal education age 6 and appendicular lean mass index in women, and FMI in men
Lahmann (2000a) [52]	Sweden	5145	Parental Occupation (recalled)	FM% Age: 45–73	Only tested in women—inverse association with parental occupational class and FM%
Lewin (2014) [54]	France	4079	Parental Education (recalled)	FMI and FM% Age: 30–79	Significant slightly inverse association between parental education and FM% in males. Association between parental education and FM% not reported for females as insignificant. Significant associations in both males and females between parental education and FMI, with lowest FMI in highest parental education group. Stronger effect in women
Loucks (2016) [56]	USA	394	Childhood SEI (age 7)	Android Fat Age: 47	Curvilinear association between childhood SEI and android fat—those in middle tertile of android fat tended to be from the most advantaged position
Pirila (2012) [61]	Finland	158	Fathers Education (recalled)	FM% *z* score, LM, % Trunk Fat Age: 32	No direct association between father’s education and FM% *z* score. Indirect pathways through own education and physical activity levels. Similar for % Trunk Fat. Evidence of direct association between father’s education and LM
Yliharsila (2007) [72]	Finland	M: 928 F: 1075	Childhood Social Class (derived from multiple points in childhood, highest record taken)	FM%, LM Age: 61.5	Lower FM% was associated with higher social class (males *P* < 0.001, females *P* = 0.031). No results reported for social class and lean mass—assumed to be insignificant

Results are presented in narrative form due to the small number of papers looking at socioeconomic position (SEP) in childhood and body composition in adulthood, and because of heterogeneity in SEP measures and body composition outcomes, preventing direct comparisons between studies.

*M* male, *F* female, *SEP* socioeconomic position, *FM* fat mass, *FMI* Fat Mass Index, *ASMI* Appendicular Skeletal Muscle Index, *LM* lean mass.

### Total body fat measures

Five papers reported on SEP in childhood and measures of fat in adulthood, testing eight different associations. In four papers, testing seven associations, greater socioeconomic advantage in childhood was associated with lower FM index (FMI) or FM% in adulthood [33, 52, 54, 72]. One study reported that the association was somewhat explained by own SEP in adulthood [33], with the others not assessing this. The fifth paper found father’s education not to be directly related to a standardised FM% score (*z*-FM%) at age 32, but reported an indirect relation through own education and current physical activity levels [61].

### Total body fat-free measures

Three papers reported on SEP in childhood and fat-free measures in adulthood, testing five associations. Two papers found evidence of positive associations, with greater socioeconomic advantage in childhood being related to higher LM [61] and higher ASM index (ASMI) [33] in adulthood. One of these presented only sex-stratified analyses and found the association with ASMI to be stronger in males before adjusting for FMI, whilst stronger in females after the adjustment for FMI [33]. The final paper reported no association with LM [72].

### Ratio and distribution measures

Three associations across three papers investigated central fat. One found an inverse association in females only [26], one a curvilinear relation, where those in the medium third of android fat had the most advantaged SEP in childhood [56]. The final paper found no association [61].

Two papers reported on android-to-gynoid ratio, presenting four associations. Both papers found greater socioeconomic advantage in childhood to be associated with lower android-to-gynoid ratio. In one paper this was only the case in females [26], whilst in the other, the association was stronger in females than males [33]. The latter paper found that the association remained with adjustment for SEP in adulthood [33].

### Adult socioeconomic predictors of adult body composition

There were 46 papers reporting on the association between SEP in adulthood and body composition in adulthood. Sixteen reported only in females, one only in males and the rest in both males and females.

### Total body fat measures

Table 4 provides a summary of the patterns reported for each fat measure. There were 75 associations tested across 30 papers. Nearly half of associations between SEP and fat measures reported were inverse (44%). Similar numbers reported non-linear (17%), made up of heterogeneous and curvilinear associations, as reported positive (11%) patterning, and almost a third of papers reported no association (28%). There were clear differences between findings in HICs and MICs. In HICs (*n* = 54), associations were predominantly inverse (59%) with no positive associations observed. In MICs (*n* = 21), most studies found positive associations (38%) or no association (43%), with only 5% reporting inverse associations.

**Table 4:** Summary of associations between socioeconomic position and fat measures in adults in high-income countries and middle-income countries.

SEP indicator	Direction of relation between SEP and fat measure												Total
	Positive			Negative			Non-linear			No relation			
	*N*	%	References	*N*	%	References	*N*	%	References	*N*	%	References	*N*
*High-income countries*													
Fat mass (kg)													
Education	0	–	–	2	50	[31]^F^ [28]	1	25	[67]	1	25	[48]	4
Composite SEP	0	–	–	1	100	[28]	0	–	–	0	–	–	1
Area-level SEP	0	–	–	3	100	[43]^F^ [37]^F^ [28]	0	–	–	0	–	–	3
Father’s education	0	–	–	1	100	[53]	0	–	–	0	–	–	1
Fat mass %													
Composite SEP	0	–	–	1	33	[64]^F^	0	–	–	2	66	[64]^M^ [71]	3
Education	0	–	–	7	58	[70]^F^ [54]^F^ [38]^F^ [67]^F^ [52, 61, 65]	2	16	[70]^M^ [54]^M^	3	25	[38]^M^ [68]^M^ [67]^M^	12
Occupational social class	0	–	–	4	66	[51]^F^ [52]^F^ [72]^M^ [72]^F^	0	–	–	2	33	[51]^M^ [68]^M^	6
Income	0	–	^–^	1	100	[65]	0	–	–	0	–	–	1
Area-level SEP	0	–	–	2	66	[54]^F^ [54]^M^	0	–	–	1	33	[42]	3
Miscellaneous	0	–	–	3	50	[54]^F^ [54]^M^ [53]	1	17	[54]^M^	2	33	[54]^F^ [68]^M^	6
Fat Mass Index													
Education	0	–	–	2	50	[33]^F^ [33]^M^	2	50	[54]^F^ [54]^M^	0	–	–	4
Income	0	–	–	0	–	–	2	100	[33]^F^ [33]^M^	0	–	–	2
Miscellaneous	0	–	–	5	63	[54]^F^ [54]^M^ [54]^M^ [33]^F^ [33]^M^	2	25	[54]^F^ [54]^M^	1	13	[54]^F^	8
*Middle-income countries*													
Fat mass (kg)													
Education	0	–	–							1	100	[34]^F^	1
Composite SEP	1	100	[60]^M^	0	–	–	0	–	–	0	–	–	1
Type of employment	0	–	–	0	–	–	1	100	[50]^F^	0	–	–	1
Fat mass %													
Composite SEP	2	100	[57]^F^ [60]^M^	0	–	–	0	–	–	0	–	–	2
Education	1	17	[39]	1	17	[46]^F^	0	–	–	4	67	[45]^M^ [45]^F^ [69]^F^ [29]^F^	6
Occupational social class	0	–	–	0	–	–	0	–	–	1	100	[45]^F^	1
Income	0	–	–	0	–	–	2	100	[39] [69]^F^	0	–	–	2
Material lifestyle	2	100	[45]^M^ [45]^F^	0	–	–	0	–	–	0	–	–	2
Fat Mass Index													
Education	1	50	[46]^F^	0	–	–	0	–	–	1	50	[63]	2
Income	0	–	–	0	–	–	0	–	–	1	100	[63]	1
Composite SEP	1	50	[60]^M^	0	–	–	0	–	–	1	50	[63]	2
*Overall distribution of* *associations—fat measures combined*													
Combined SEP		11%			44%			17%			28%		75
HIC combined SEP		0%			59%			19%			22%		54
MIC combined SEP		38%			5%			14%			43%		21

Positive associations indicate an increase in fat measure with an increase in socioeconomic advantage; inverse associations indicate a decrease in fat measure with an increase in socioeconomic advantage; non-linear associations indicate that the association between SEP and fat measures is either curvilinear or heterogeneous. Miscellaneous SEP measures are where less than two papers reported on the measure. Total *N* represents the total number of reported associations between the given SEP measure and the body composition measure (i.e., total number of associations reporting on education and FM). There may be two associations from one paper per SEP measure, if only gender-stratified data are presented. The *N* in the ‘direction of relation’ groups (i.e., positive, negative, non-linear, no association) refers to the number of associations reporting each patterning within the given SEP measure and body composition measure combination (i.e., number of positive associations reported between education and FM), and corresponds to the references included. The % in the ‘direction of relation’ groups indicates the number of associations reporting a particular patterning (i.e., positive) as a percentage of the total number of associations for the given SEP measure and body composition measure (i.e., education and FM).

^F^Indicates results for females only.

^M^Indicates results for males only.

FM% was used 44 times across 21 papers, FMI was used 19 times across 10 papers and FM 12 times across 10 papers. Each fat measure showed predominantly inverse associations, and this was driven by inverse associations in HICS. In HICs, inverse associations were reported more frequently for FM (78%) compared to FM% (58%) and FMI (50%). Non-linear associations (including both heterogeneous and curvilinear) were more frequently reported for FMI (43%) than for other measures. In MICs, FM% and FMI predominantly showed positive associations (38% and 40%, respectively) and no association (38% and 60%, respectively). FM was used only three times in MICs with all patterns of association being different.

For education, the most commonly studied SEP measure, 12 of 29 associations showed an inverse association with a measure of fat (41%), again driven by findings in HICS. In MICs, education showed predominantly no association (six out of nine associations). Inverse associations were predominantly observed for occupational social class (six out of ten), but the measure was used almost exclusively in HICs. For area-level SEP, which was used only in HICs, predominantly inverse associations (six out of eight) were reported. In contrast, four out of nine associations with composite SEP showed positive associations. All of those reporting positive associations were conducted in MICs. Four of the remaining five studies that showed either inverse or no association were conducted in HICs. Non-linear associations were most frequently reported in studies investigating income in both HICs and MICs (in two out of three associations in both).

In studies that presented sex-stratified or sex-specific analysis (66 associations across 22 papers), inverse associations were reported over twice as frequently in females (77%) compared to males (33%) in HICs. There was a higher proportion of non-linear associations in males (29%) compared to females (14%) (Supplementary Fig. 1). In studies from MICs, no association was more commonly reported in women (57%) compared with men (44%). Positive associations were more commonly reported in men (55%) than women (21%).

### Total body fat-free measures

Table 5 provides a summary of the patterns identified for each of the different fat-free measures. There were 44 associations tested across 19 papers. The majority found no association between SEP and fat-free measures (55%), whilst 32% found evidence of positive associations, 7% found inverse and 7% found non-linear associations. More associations were tested in HICs (30 association across 14 papers) compared to MICs (14 associations across 5 papers), and patterns of association were similar in both settings.

**Table 5:** Summary of associations between socioeconomic position and lean measures in adults in high-income countries and middle-income countries.

SEP indicator	Direction of relation between SEP and lean measure												Total
	Positive			Negative			Non-linear			No relation			
	*N*	%	References	*N*	%	References	*N*	%	References	*N*	%	References	*N*
*High-income countries*													
Fat-free mass													
Education	2	50	[66]^F^ [66]^M^	0	–	–	0	0	^–^	2	50	[67]^F^ [67]^M^	4
Miscellaneous	0	–	–	1	50	[27]^F^	0	0	–	1	50	[27]^M^	2
Lean mass													
Education	2	33	[70]^F^ [31]^F^	0	0	–	0	0	–	4	66	[70]^M^ [30]^F^ [48] [61]	6
Miscellaneous	1	33	[53]	0	0	–	1	33	[30]^F^	1	33	[37]^F^	3
Lean mass %													
Father’s education	1	100	[53]	0	–	–	0	–	–	0	–	–	1
Lean Mass Index													
Occupational social class	0	0	–	1	50	[27]^F^	0	0	–	1	50	[27]^M^	2
Appendicular skeletal muscle													
Education	0	0	–	0	0	–	0	0	–	2	100	[47]^M^ [47]^F^	2
Income	0	0	–	0	0	–	1	50	[47]^M^	1	50	[47]^F^	2
Appendicular Skeletal Muscle Index													
Education	1	25	[33]^F^	0	0	–	0	0	–	3	75	[41]^F^ [49]^F^ [33]^M^	4
Income	2	100	[33]^F^ [33]^M^	0	0	–	0	0	–	0	–	–	2
Miscellaneous	1	50	[33]^F^	0	0	–	0	0	–	1	50	[33]^M^	2
*Middle-income countries*													
Fat-free mass													
Education	2	50	[32]^M^ [34]^F^	1	13	[32]^F^	0	0	^–^	0	–		3
Composite SEP	1	33	[60]^M^	0	–		0	0	–	0	–		1
Fat Free Mass Index													
Composite SEP	0	–	–	0	–	–	0	–	–	1	100	[60]^M^	1
Lean mass													
Employment type	0	–		0	0	–	1	50	[50]^F^	0	–		1
Appendicular skeletal muscle													
Education	1	20	[32]^F^	0	0	–	0	0	–	2	–	[32]^M^ [63]	3
Income	0	0	–	0	0	–	0	–		1	100	[63]	1
Employment status	0	0	–	0	0	–	0	0	–	1	100	[63]	1
Appendicular Skeletal Muscle Index													
Education	0	0	–	0	0	–	0	0	–	1	100	[63]	1
Income	0	0	–	0	0	–	0	0	–	1	100	[63]	1
Employment status	0	0	–	0	0	–	0	0	–	1	100	[63]	1
*Overall distribution of* *associations—fat measures combined*													
Combined SEP		32%			7%			7%			55%		44
HIC combined SEP		33%			7%			7%			53%		30
MIC combined SEP		29%			7%			7%			57%		11

Positive associations indicate an increase in fat-free measure with an increase in socioeconomic advantage; inverse associations indicate a decrease in fat-free measure with an increase in socioeconomic advantage; non-linear associations indicate that the association between SEP and fat-free measures is either curvilinear or heterogeneous. Miscellaneous SEP measures are where less than two papers reported on the measure. Results for Pirila are reported as LBM but have been included here with FFM due to similarity of measure. Total *N* represents the total number of reported associations between the given SEP measure and the body composition measure (i.e., total number of associations reporting on education and FFM). There may be two associations from one paper per SEP measure, if only gender-stratified data are presented. The *N* for the direction of relation groups (positive, negative, non-linear, no association) refers to the number of associations reporting each patterning within the given SEP measure and body composition measure combination (i.e., number of positive associations reported between education and FFM), and corresponds to the references included. The % for the direction of relation groups indicates the number of associations reporting a particular patterning (i.e., positive) as a percentage of the total number of associations for the given SEP measure and body composition measure (i.e., education and FFM).

^a^Indicates study conducted in a MIC.

^F^Indicates results for females only.

^M^Indicates results for males only.

LM was used 10 times across 8 papers while FFM, ASM and ASMI were used 9–11 times each across 3–6 papers. Percentage LM (LM %), LM index (LMI) and FFM index (FFMI) were used in one paper each. FFM showed a greater frequency of positive associations (50%) while for all other measures no association was most commonly observed. ASMI reported a higher number of positive associations (36%) compared to LM and ASM. LM and ASM found positive associations in 10–30% of associations. Inverse and non-linear patterns were reported in approximately 0–20% of the associations for LM, FFM, ASM and ASMI.

For measures that include bone (FFM and LBM) (*n* = 11), there was a higher proportion of positive associations reported (45%) compared to measures that did not include bone, i.e., LM (*n* = 33, 27%). There was little difference in the distribution of associations between those that used index or percentage measures compared to those that used raw measures. Two papers made additional adjustments [33, 66], for either FM or body size that resulted in associations reversing in direction to become positive, or increasing in strength.

For all measures of SEP, no associations were most frequently observed. Positive associations were more frequently reported for education (35% of 23 associations) and income (29% of 7) compared with social class (17% of 6). Income showed a higher percentage of non-linear associations (29%) and a lower number of no associations (43%) compared to all other SEP measures. Composite measures of SEP were used twice and area-level SEP once.

Among studies that presented sex-specific or sex-stratified analysis (46 associations across 16 papers), there was greater variability in the patterns of associations observed among women in HICs compared with men in both HICs and MICs and women in MICs (Supplementary Fig. 2). There were a higher percentage of positive (40%) and inverse (16%) relationships, and a lower percentage reporting no association (40%). Among men and women in MICs and men in HICs, there was more consistent evidence of no association across the studies (70–73%).

### Ratio and distribution measures

Thirteen papers reported on the association between SEP and ratio or distribution measures (Supplementary Table 2). Only two were from MICs preventing comparison of differences by the income level of the country.

There were 14 associations across ten papers that investigated the association between SEP and a measure of central fat. Four papers used the same all-female sample (SWAN) [40, 44, 55, 58], and one additional paper also tested the association in women only [50]. The majority of associations (9 out of 14, 64%) reported that greater socioeconomic advantage was associated with lower central adiposity. For education, five out of the seven associations found evidence that lower education level was associated with greater central fat, whilst two out of the three papers using income found some evidence of higher central fat among those with lower income.

Two papers used a distribution measure other than central fat. One found women who engaged in more labour-intensive occupations had lower leg FM [50]. The other found no association between education and the distribution of either upper or lower SMM [32].

Six associations across three papers reported on ratios, one conducted in an all-female population. One paper, which tested the association using education, occupational social class and income, found that greater socioeconomic advantage was related to a lower android-to-gynoid ratio [33]. Men showed a stronger association for education compared to other SEP measures, and also a stronger association than that observed in women when measured by education, whilst a heterogeneous association was observed for occupational social class [33]. Another paper found that those with higher education level were more likely to have a normal compared to high ratio of fat to LM, whilst employment was related to the ratio in a non-linear fashion [62]. In the third study no association was found between income and the ratios of central fat types in females [44].

## Discussion

This systematic review finds evidence of socioeconomic inequalities in body composition, although the direction and strength of these inequalities varies by measure of SEP, measure of body composition, sex and economic development of the country of study. The review generally finds evidence of associations between more advantaged SEP in both childhood and adulthood and lower levels of total body FM, particularly in HICs, irrespective of which SEP or FM measure is used. Such associations were more frequently observed among females than males. In MICs, the majority of studies showed positive or no association for fat measures. For fat-free measures, findings were more mixed, with the majority of papers reporting no association or greater socioeconomic advantage being associated with greater FFM. There is some evidence of similar positive associations between childhood SEP and adult fat-free measures in HICs, although the small number of studies means caution is required in interpretation of these findings. No studies reported associations for childhood SEP and adult body composition in MICs, and no studies conducted in LICs were identified.

Our findings for adult SEP and fat measures are consistent with previous systematic reviews, showing predominantly inverse associations for anthropometric measures of adiposity such as BMI and waist circumference in HICs, especially among women [2, 3]. Also similar to reviews based on BMI and other anthropometric measures [2, 3, 7], we find positive associations to only be observed in MICs. We also found SEP to be inversely related to measures of central fat, whilst evidence for other measures of fat distribution is too sparse to draw conclusions [3].

Our findings in relation to childhood SEP and adult body fat measures are also similar to those for BMI that find mostly inverse associations [4, 6]. However, the small number of studies means that conclusions regarding differences between males and females are not possible. No studies reported on the association in MICs, and it was therefore not possible to compare findings between MICs and HICs. To the best of our knowledge, no systematic reviews have previously considered social inequalities in measures of FFM. One review did find more consistent evidence than our review that advantaged SEP in childhood is related to better physical capability in adulthood [76], with such measures of physical capability (i.e., grip strength) being correlated with muscle mass and strength [77, 78]. Few studies tested if associations with childhood SEP were independent of adult SEP, which has implications for the lasting role of early development in nutrition and physical activity patterns [79, 80]. Consideration of both child and adult SEP would also allow investigation of latency, trajectory or accumulation effects of SEP across the life course and help to identify the best time to intervene to prevent inequalities in body composition.

A range of measures of FM and FFM were used across the studies, and the majority of papers used a raw measure of mass, rather than measures indexed to height. However, indexed measures of FM and FFM are considered to be more appropriate as they can be interpreted independently of the other, and account for the contribution of height to mass [81, 82]. We found stronger evidence of inverse association for fat when using the raw measure, rather than indexed measures, indicating less evidence of inequalities in fat when appropriately accounting for height.

For fat-free measures, there was also variation in what composition of FFM was captured. Measures attained through BIA do not separate lean from bone mass, while use of DXA does allow separation of bone. Studies that used measures that included bone mass showed a higher proportion of positive associations compared to those that did not, indicating that bone may be contributing to observed inequalities in FFM. In addition, there were inconsistencies in the adjustment of LM for FM. Previous research has shown that adaptive increases in LM occur with increases in FM, highlighting a need to consider adjustment for FM [83–85]. In this review, adjusting FFM measures for FM or body size resulted in the direction of association reversing, becoming positive [33] or existing positive associations increasing in strength [66].

Differences observed in associations according to SEP measures could largely be explained by differences between HICs and MICs. Both occupational social class and area-level measures of SEP were predominantly used in HICs and also reported the highest proportion of inverse relations with total FM. Composite measures of SEP were more frequently used in MICs and reported a higher proportion of positive associations with total FM. Income was the only measure used in HICs that showed a low number of inverse associations and a high number of non-linear associations for FM measures. Income is considered a direct measure of material resources and is most prone to short-term change [8], which may explain the greater observed heterogeneity. Education is a more stable measure of SEP that captures early life conditions whilst also a determinant of later life SEP and reflects knowledge assets and health literacy as well as health behaviours [8]. Similarly, occupational social class is a good overall measure of SEP as it captures aspects of an individual’s education, income, social standing in addition to their occupation [8]. It is possible that area-level SEP may also be a particularly strong predictor of inverse associations in HICs due to the close link with obesogenic elements in the environment [86, 87].

Differences in findings between HICs and MICs, particularly for FM, may be, at least in part, explained by the nutrition transition. Consumption of energy-dense food that is high in fats and sugars is related to higher adiposity, whilst protein and micronutrients are required for lean tissue development. As countries develop, food becomes more abundant and accessible and, in particular, more frequently characterised by high-energy-dense and calorific foods [88]. In MICs, those with greater socioeconomic advantage have greater food security and access to the high-energy-dense foods, and more calorific diets [89]. In HICs, high-energy-dense and calorific foods tend to be cheaper and consumed more frequently among individuals of less-advantaged SEP [90, 91].

Physical activity is an important determinant of LM development and maintenance, as well as being important for maintenance of healthy adiposity levels. Levels of physical activity between MICs and HICs may be affected by different timings in the onset of the obesogenic environment and nutrition transition [88], which is in part characterised by a shift from more labour-intensive lifestyles to more sedentary lifestyles [92]. Although those in less-advantaged socioeconomic circumstances are more likely to work manual jobs, and therefore have higher occupational physical activity, those in positions of socioeconomic advantage in HICs tend to participate in more leisure time physical activity compared to those in less-advantaged positions [93, 94]. In particular, there is evidence of greater vigorous activity from leisure time activities among those in more advantaged positions in HICs [94]. This, combined with differences in nutrition, may explain the existence of inverse associations for fat measures and positive associations for lean measures in HICs, where those with low fat are also leaner. The existence of predominantly positive associations between SEP and fat measures and to a lesser extent in lean measures in MICs may reflect that those in less-advantaged SEPs with low fat may be malnourished, and therefore also have less muscle.

Differences in association by sex observed for both lean and fat measures, with women in HICs most likely to experience inequalities in lean and fat measures compared to other groups, may be explained by increases in gross national product shifting the burden of obesity to those of a less-advantaged position within society, affecting women first [95]. This may be a result of the greater pressures of weight-related ideals faced by women, which are easier to maintain in a position of advantage [96].

Based on previous evidence using anthropometric measures, it is possible that there are differences in inequality according to ethnicity. Among HICs, minority ethnic groups tend to have higher prevalence of obesity compared to the majority [97] and are more likely to be living in disadvantaged circumstances [98]. In the US, whilst a positive association among income and obesity is observed among African American and Caribbean Black men, an inverse association is observed for women [99]. In the UK, there is evidence that increased acculturation results in convergence in obesity among minority ethnic groups, all except for Black Caribbean groups [100]. Few studies in the review considered analyses stratified by ethnicity and further research is needed to understand the complexity of ethnic differences in body composition inequalities in both HICs and MICs, which are likely to be complex, influenced by cultural factors, migration status and structural racism.

### Strengths and limitations

This review was registered with PROSPERO and has been carried out according to the published protocol [20]. Bias in the process was minimised by two independently working reviewers conducting each stage of the review, including selection of studies and extraction of data.

The review has an inclusion and exclusion criteria capturing a broad range of evidence, thereby reducing selection bias. However, this resulted in large heterogeneity among the included papers in both exposures, outcomes and statistical reporting with multiple associations often being tested in a single paper. This variation in statistical analyses prevented a meta-analysis from being conducted. Our narrative review followed previous similar reviews in using the association, rather than the paper, as the unit of analysis. However, this meant that in some cases one paper contributed multiple associations to the overall percentages. The same data sets were also used in multiple papers.

We assigned associations to the four categories of association using the effect estimates and confidence intervals, which convey more about the direction and strength of effect, and the accuracy of these estimates [101, 102]. This is to overcome the limitations where there has been a heavy reliance on *P* values to convey statistical results [102, 103]. However, many papers included did rely on *P* values in the reporting of their results and thus there may have been selective reporting of significant findings.

There is inconsistency in the literature relating to the terminology used to describe FFM [104], with a small number of papers using incorrect terminology based on the description of their body composition measure. Despite best efforts to ensure comparability by applying standard definitions of fat-free measures across the review, some papers did not provide enough clarity on the measures used to do this confidently, i.e., such as clearly stating if bone is included or excluded in estimates of FFM. This highlights the need for consistent definitions to be applied and used across the body composition literature, and for authors to provide clarity on the measures used, specifically ensuring use of the correct term if bone is included (FFM or LBM) or excluded (LM) and using appropriate and consistent terminology throughout when measures have been indexed or converted to percentages. We were unable to assess the differences in association by birth year due to lack of information. We were also not able to assess differences between childhood SEP and adulthood body composition by age and sex and income level of country due to the small number of papers reporting such associations.

### Implications and conclusion

The results of this review indicate that inequalities in BMI are likely to capture inequalities in FM in adults, but not inequalities in FFM for which we find weak evidence of associations. Compared to total body FM, few studies looked at measures of FFM that exclude bone and that are indexed to height. There is a need for research to adopt better and more consistent measures of FFM that account for the contribution of height and bone, in order to better understand inequalities in LM. This may be particularly important for research in older age, as muscle mass becomes increasingly important for functional outcomes.

Few studies investigated the association between childhood SEP and adulthood body composition, and none were in MICs. Childhood SEP has previously been shown to be a strong predictor of BMI in adulthood, and further research is needed to understand how disadvantage may accumulate over the life course and influence body composition in adulthood. No data were available from LICs across the whole review, an area that warrants further research. Only a small number of studies provided birth years of participants, preventing investigation of secular differences in inequalities in body composition in the context of persisting inequalities in BMI. The majority of studies were conducted in those aged 40 and above, with fewer studies looking at young to mid-adult life, preventing full assessment of difference in body composition across the adult life course. Follow-up of cohorts across adulthood are needed to identify if there are either secular changes in body composition or age-related changes in body composition, or the existence of both simultaneously and how this might influence social inequalities.

The differences in associations between SEP and FM between HICs and MICs indicate emerging and, in some cases, reversing inequalities in body composition as countries go through the nutrition transition and with the onset of the obesogenic environment. These findings suggest that action is required in MICs to mitigate the negative effects of this transition. Mitigating action is likely needed in LICs also, which are expected to be further behind in the nutrition transition than MICs, and so information in LICs is needed. In particular, efforts should continue to focus on reducing the abundance of cheap energy-dense food in poorer communities and ensure access to healthy and nutritious food across SEP groups, as a way to combat inequalities in FM. Attention should also be paid to promotion of physical activity to ensure healthy levels of FFM across all SEP groups, and especially into older age where muscle mass may be more important for metabolic and functional outcomes.

## Supplementary information

Supplementary File 1 – PRISMA 2009

Supplementary File 2

Supplementary Table 1

Supplementary Figure 1 Fat mass

Supplementary Figure 2 Fat free mass

Supplementary Table 2
