# Supplementary material for: Life course socioeconomic position and body composition in adulthood: a systematic review and narrative synthesis
Source: Int J Obes (Lond). 2021 Jul 27;45(11):2300–15. doi: 10.1038/s41366-021-00898-z (PMC8528709; doi:10.1038/s41366-021-00898-z)
Supplement: Supplementary file 3 — Supplementary Table 1 [file 41366_2021_898_MOESM3_ESM.docx]

| Body Composition Measures Used | Abbreviations | Description |
| --- | --- | --- |
| Fat Mass Measures | | |
| *Raw Measures* | | |
| Fat Mass (kg)  (or Body Fat (kg)) | FM  (or BF) | Total mass of fat measured using BIA/DXA or other  Sometimes used interchangeably with body fat in the papers. |
| *Indexed Measures* | | |
| Fat Mass Percentage  (or Body Fat Percentage) | FM%  (or BF%) | Total fat mass as a percentage of total body mass:  *FM% = (fat mass (kg) / total body mass (kg)) x 100*  Sometimes used interchangeably with BF%. |
| Fat Mass Index (kg/m^2^) | FMI | Total mass of fat indexed to height squared:  *FMI = fat mass (kg) / height (m)^2^* |
| Lean Mass Measures | | |
| *Raw Measures* | | |
| Fat-Free Mass (kg)* | FFM | The difference between total body weight and FM.  *FFM = total body mass (kg) – fat mass (kg)*  Includes bone mass. Most frequently calculated from BIA which is not able to separate bone from total body mass. |
| Lean Body Mass (kg) | LBM | LBM is similar to FFM, but also includes additional essential fat found in the nervous system, cell membranes and bone marrow, not captured by FFM [1].  *LBM = FFM (kg) + essential fats*  LBM is often considered synonymously, and used interchangeably, with FFM. Differences between LBM and FFM are around 2-10% [2].  Most commonly measured using BIA. |
| Lean Mass (kg) | LM | Total body mass excluding fat and bone tissue ^$^.  *LM =* *total body mass (kg) – (fat mass (kg) + bone mass (kg))*  Most frequently calculated through DXA as able to distinguish bone mass. “Lean mass”, if not measured by DXA, is more likely referring to LBM or FFM. Where the term lean body mass is used in relation to a measurement using DXA, we include as LM as it is probable that it excludes bone. |
| Appendicular Skeletal Muscle (kg) | ASM | Muscle mass of the four limbs without fat and bone tissue.  Appendicular skeletal muscle represents 75% of total skeletal muscle [3, 4].  In some cases ASM is used interchangeably with muscle mass (MM) or appendicular MM. |
| *Indexed Measures* | | |
| Fat-Free Mass Percentage | FFM% | Total fat-free mass as a percentage of total body mass:  FFM% = (FFM (kg) / total body mass (kg)) x 100 |
| Fat-Free Mass Index (kg/m^2^) | FFMI | Total fat-free mass indexed to height squared:  *FFMI = FFM (kg) / Height (m) ^2^* |
| Lean Mass Percentage | LM% | Total lean mass as a percentage of total body mass:  LM% = (LM (kg) / total body mass (kg)) x 100 |
| Lean Mass Index (kg/m^2^) | LMI | Total lean mass indexed to height squared:  *LMI = LM (kg) / Height (m) ^2^* |
| Appendicular Skeletal Muscle Percentage | ASM% | Appendicular Skeletal Muscle as a percentage of total body mass:  ASM% = (ASM (kg)/ total body mass (kg)) x 100 |
| Appendicular Skeletal Muscle Index (kg/m^2^) | ASMI | Total appendicular skeletal muscle indexed to height squared:  *ASMI = ASM (kg) / Height (m) ^2^* |

**Supplementary Table 3. Description and abbreviations of direct body composition measures identified in the review**

**Footnotes.** In some cases measures that are indexed to height^2^ may be calculated with an alternative power due to high correlation of the indexed measure with height. ^$^Variations in the way LM may be calculated: exclusion of the head from estimates due to high proportion of bone meaning estimates of soft tissue are less accurate; participants observe fasting conditions and take no exercise prior to measurement; lean body mass may also be calculated as “dry lean mass” if water content is also subtracted from estimate.

1. Janmahasatian, S., et al., *Quantification of lean bodyweight.* Clin Pharmacokinet, 2005. **44**(10): p. 1051-65.

2. Scafoglieri, A. and J.P. Clarys, *Dual energy X-ray absorptiometry: gold standard for muscle mass?* J Cachexia Sarcopenia Muscle, 2018. **9**(4): p. 786-787.

3. Hansen, R.D., et al., *Determination of skeletal muscle and fat-free mass by nuclear and dual-energy x-ray absorptiometry methods in men and women aged 51-84 y (1-3).* Am J Clin Nutr, 1999. **70**(2): p. 228-33.

4. Buckinx, F., et al., *Pitfalls in the measurement of muscle mass: a need for a reference standard.* J Cachexia Sarcopenia Muscle, 2018. **9**(2): p. 269-278.
