# Supplementary material for: Life course socioeconomic position and body composition in adulthood: a systematic review and narrative synthesis
Source: Int J Obes (Lond). 2021 Jul 27;45(11):2300–15. doi: 10.1038/s41366-021-00898-z (PMC8528709; doi:10.1038/s41366-021-00898-z)
Supplement: Supplementary file 4 — Supplementary Figure 1 Fat mass [file 41366_2021_898_MOESM4_ESM.docx]

**Supplementary Figure 1. Distribution of associations for fat measures by gender and income level**
Females in HICs (N=22): 77% inverse associations, 0% positive associations, 14% non-linear associations, 9% show no association; Males in HICs (N=21): 33% inverse associations, 0% positive associations, 29% non-linear associations, 38% show no association; Females in MICs (N= 14) 7% inverse associations, 21% positive associations, 14% non-linear associations, 57% show no association; Males in MICs (N= 9) 0% inverse associations, 56% positive associations, 0% non-linear associations, 44% show no association.
