# Supplementary material for: Life course socioeconomic position and body composition in adulthood: a systematic review and narrative synthesis
Source: Int J Obes (Lond). 2021 Jul 27;45(11):2300–15. doi: 10.1038/s41366-021-00898-z (PMC8528709; doi:10.1038/s41366-021-00898-z)
Supplement: Supplementary file 5 — Supplementary Figure 2 Fat free mass [file 41366_2021_898_MOESM5_ESM.docx]

**Supplementary Figure 2. Distribution of associations for fat-free measures by gender and income level**
Females in HICs (N=15): 13% inverse associations, 40% positive associations, 7% non-linear associations, 40% show no association; Males in HICs (N=10): 0% inverse associations, 20% positive associations, 10% non-linear associations, 70% show no association; Females in MICs (N= 11) 9% inverse associations,9% positive associations, 9% non-linear associations, 73% show no association; Males in MICs (N= 10) 0% inverse associations, 30% positive associations, 0% non-linear associations, 70% show no association.
