# Supplementary material for: Life course socioeconomic position and body composition in adulthood: a systematic review and narrative synthesis
Source: Int J Obes (Lond). 2021 Jul 27;45(11):2300–15. doi: 10.1038/s41366-021-00898-z (PMC8528709; doi:10.1038/s41366-021-00898-z)
Supplement: Supplementary file 6 — Supplementary Table 2 [file 41366_2021_898_MOESM6_ESM.docx]

**Supplementary Table 4. Results of associations between socioeconomic position and adult ratio and distribution measures**

| Paper | Country | N | Age | Study or Description of the Population | SEP Measure | Body Composition measure | Findings |
| --- | --- | --- | --- | --- | --- | --- | --- |
| Kazlauskaite (2012)  [43] | USA | 257 | 52 | SWAN  (females only) | Income | Log IAT, IAT:SAT Ratio | No association between income and Log IAT or with IAT:SAT ratio in all three models. Model 1 adjusted for risk groups (ethnicity, menopausal status), income, age and percent body fat.  Model 2 = Model 1 plus low physical activity (Kaiser Physical Activity Survey score) and smoking status  Model 3 = Model 2 plus energy intake ethnicity interaction |
| Loucks (2015)  [55] | USA | 394 | 46-48 | New England Family Study | Education | Android FM | Highest percentage of individuals with low education (% with less than high school education) in highest android fat tertile. |
| Beydoun (2009)  [34] | USA | 1,227 | 30-64 | HANDLS | SEP | Trunk Fat, Trunk fat as % of total body fat | Association differed by gender and ethnicity. SEP was inversely associated to measures of central adiposity in white American women, and positively associated in African American women. There was no association between SEP and central adiposity in white American or African American men. |
| Pirila (2012) [60] | Finland | 158 | 32 | Sample taken from birth cohort of full-term babies born in Helsinki in 1975. | Education | % Trunk fat | Decrease in % Trunk fat with an increase in education level (r=-0.147, p=0.07). |
| Bhupathiraju (2011)  [35] | USA | F: 465  M: 164 | 54-75 | Boston Puerto Rican Osteoporosis Study | Education, Income | Abdominal Fat | Significant difference between fat tertiles for those with the least and most education in women. Lowest percentage with low education (<9th grade) and highest percentage with high education (at least some college) in lowest fat tertile. No significant difference between fat groups for percentage with medium education.  Income inversely related to abdominal fat in women, with increase in central adiposity with increase in income (P=0.02).  No association between education or income with abdominal fat in men. |
| Dugan (2010)  [39] | USA | 369 | 50.7 | SWAN  (females only) | Education | IAT | Little evidence of an association between education and IAF (Beta: 7.1, SE= 6.5, P=0.28) |
| Mongraw-Chaffin (2017)  [58] | USA | 1,910 | 45-85 | MESA | Education, Income | Visceral Fat | Greater percentage of those with most advantaged education or income level in the lowest visceral fat tertile (P=0.001 and P=0.055 respectively. Cuzick non-parametric test). |
| McClure (2011)  [57] | USA | 301 | 46-58 | SWAN  (females only) | Education, Financial Strain | Visceral Fat | No association between education or financial strain and visceral fat. |
| Lewis (2009) [54] | USA | 418 | 42-61 | SWAN  (females only) | Education | Visceral Fat | Weak inverse association between years in education and visceral fat (B =-1.31, Beta=-0.05, SE=0.99, P=0.19) |
| Kulkarni (2019)  [49] | India | 278 | 41 | Adult women who were not pregnant or lactating, residing in a large urban slum (Addagutta) in Hyderabad. | Occupation Type | Trunk FM, Leg FM | Trunk FM and leg FM were lower among sweepers (Beta= -554, p=0.009 and Beta = -587, p=0.002, respectively) and construction workers (Beta=-506, p=0.013 and Beta=-548, p=0.003, respectively) compared to home makers. Leg FM was also lower in servant maids (Beta=-354, p=0.036). Trunk FM and leg FM did not significantly differ in beedi makers compared to home makers. |
| Powell (2016)  [61] | Italy | 3,441 | 18-81 | Participants selected from ongoing cohort in Milan | Education, Employment | VAT:FFMI, FM:FFM, | Higher percentage with university degree and lower percentage with elementary school in normal compared to high VAT:FFMI and FM:FFM groups (both P<0.001). Curvilinear relation for employment, with differences in VAT:FFMI and FM:FFM across employment groups (both P<0.001). |
| Bann (2014)  [32] | UK | M: 746  F: 812 | 60-64 | NSHD | Education (age 26) , Occupational Class (age 53), Household Income (age 60-64) | Android to Gynoid Ratio | Inverse associations between all SEP measures and android to gynoid ratio in both males and females (SII ranges from 3.26 to 8.42 and p values range from <0.01 to 0.04), with exception for occupational class in males, where deviation from linearity indicates a heterogeneous association (SII: 3.81; 95% CI: −0.33 to 7.96; p=0.07). Significant sex interaction for association between education and android to gynoid ratio (p=0.05). |
| Bai (2016)  [31] | China | 212 | 60-99 | Men and women recruited through printed advertisement from the health survey centre of Shanghai Huadong Hospital. | Education | Upper limb skeletal muscle mass; lower limb skeletal muscle mass | Education a non-significant predictor of upper and lower limb skeletal muscle mass. |

**Footnotes.** F- Female; M – Male; SEP – Socioeconomic Position; SII – Slope of Inequality Index; FM - Fat Mass; FFM - Fat Free Mass; FFMI – Fat Free Mass Index; IAT – intra-abdominal adipose tissue; SAT – subcutaneous abdominal adipose tissue; VAT – visceral adipose tissue. SWAN- The Study of Women's Health Across the Nation; HANDLS- Healthy Aging in Neighbourhoods of Diversity across the Life Span; NSHD- National Survey of Health and Development.
